# Chemometric Analysis of the Amino Acid Requirements of Antioxidant Food Protein Hydrolysates

**DOI:** 10.3390/ijms12053148

**Published:** 2011-05-13

**Authors:** Chibuike C. Udenigwe, Rotimi E. Aluko

**Affiliations:** The Department of Human Nutritional Sciences and the Richardson Centre for Functional Foods and Nutraceuticals, University of Manitoba, Winnipeg, MB R3T 2N2, Canada; E-Mail: umudenig@cc.umanitoba.ca

**Keywords:** antioxidant prop1erties, free radicals, reactive oxygen species, chemometrics, partial least square regression, amino acids

## Abstract

The contributions of individual amino acid residues or groups of amino acids to antioxidant activities of some food protein hydrolysates were investigated using partial least squares (PLS) regression method. PLS models were computed with amino acid composition and *3-z* scale descriptors in the *X*-matrix and antioxidant activities of the samples in the *Y*-matrix; models were validated by cross-validation and permutation tests. Based on coefficients of the resulting models, it was observed that sulfur-containing (SCAA), acidic and hydrophobic amino acids had strong positive effects on scavenging of 2,2-diphenyl-1-picrylhydrazyl (DPPH) and H_2_O_2_ radicals in addition to ferric reducing antioxidant power. For superoxide radicals, only lysine and leucine showed strong positive contributions while SCAA had strong negative contributions to scavenging by the protein hydrolysates. In contrast, positively-charged amino acids strongly contributed negatively to ferric reducing antioxidant power and scavenging of DPPH and H_2_O_2_ radicals. Therefore, food protein hydrolysates containing appropriate amounts of amino acids with strong contribution properties could be potential candidates for use as potent antioxidant agents. We conclude that information presented in this work could support the development of low cost methods that will efficiently generate potent antioxidant peptide mixtures from food proteins without the need for costly peptide purification.

## 1. Introduction

Antioxidant enzymatic food protein hydrolysates and food-derived peptides have gained particular interest as potential ingredients for formulation of functional foods and natural health products. Such formulated products could be used for human health sustenance especially for prevention and management of chronic diseases induced or propagated by oxidative stress. There is abundant information in the literature on food protein hydrolysates, peptide fractions and purified peptides with antioxidant activities in various oxidative models *in vitro* and in cell cultures. The antioxidant activity of food-derived peptides is based on scavenging of free radicals or reactive species [1,2], which is predominantly based on proton-coupled single electron or hydrogen atom transfer mechanisms [3,4]. In addition, food protein-derived antioxidant peptides also exhibit their activity by chelating pro-oxidant transition metals, e.g., Fe^2+^ and Cu^2+^, reducing ferric ion, inhibiting oxidation of biological macromolecules such as unsaturated fatty acids [1,5] and cellular regulation of gene expression of antioxidant proteins, e.g., heme oxygenase-1 and ferritin [6]. Antioxidant activities of free amino acids have been evaluated [7] and a number of amino acids have been proposed to contribute positively to the antioxidant activity of purified food-derived and synthetic natural peptides. These amino acids include *Trp*, *Tyr*, *Met*, *Cys*, *His*, *Phe* and *Pro* [1,7–10]. Till date, several studies have attributed the antioxidative properties of many food protein hydrolysates, peptide fractions and purified peptides to these amino acids, but the chemistry and mechanisms of action have not been studied in detail. It is generally agreed upon that peptides possess substantially better antioxidant activities than their parent proteins and constituent amino acids [1], possibly due to increased accessibility of the functional side chain (R-group) to the reactive species, and the electron-dense peptide bonds. However, previous studies have indicated that some amino acids may be more active than their parent peptides [11,12].

For practical cost-effective application of food-derived peptides in the formulation of health-promoting food products, important limiting factors to be considered include a combination of high yields and potency of the natural bioactive ingredients. The expensive procedures and low product yield often associated with food protein-derived peptide purification and synthesis underscores the need to develop natural enzymatic food protein hydrolysates and peptide fractions that will possess potent antioxidant activities without the need for further extensive processing. In order to achieve this goal, initial directions of approach should involve elucidation of amino acid requirements for potency of food protein hydrolysates, and development of enzymatic hydrolysis and simple processing methods for enrichment of the desired amino acid residues in the peptide mixtures based upon their unique physicochemical properties. Indeed, the presence of the proposed antioxidant amino acid residues (*Trp*, *Tyr*, *Met*, *Cys*, *His*, *Phe and Pro*) may promote the antioxidant activities of food protein hydrolysates. However, the contributions of other amino acid residues to the oxidative system and the relative contribution/interactions of the so-called antioxidant amino acids in the complex peptide mixtures remain unclear. Moreover, the specific role of food-derived peptide antioxidants can be influenced by the type of oxidative species and chemistry of the reaction medium (e.g., pH) [2,3], which influence the properties of functional amino acid side chain groups. In-depth understanding of the relationship between composition and antioxidant activity of food protein hydrolysates and peptides is desirable for directed efforts towards the discovery of safer functional food ingredients [1]. Therefore, the objective of this study was to evaluate the contributions of individual amino acid residues, groups of amino acids (based on similar physicochemical properties of the R-group) and amino acid structural properties (based on *3-z* scale amino acid descriptor) of food protein hydrolysates to antioxidant activities in four different oxidative assay systems using partial least squares (PLS) regression.

## 2. Results and Discussions

PLS modeling is a widely used descriptive and predictive chemometrics approach for quantitative structure-activity relationship (QSAR) studies to elucidate how variation of molecular structures affect bioactivity of therapeutic agents, especially when working with high number of descriptor variables compared to the number of observations [13,14]. This method has been widely applied in food science research for developing models in QSAR studies of food-derived peptides such as bitter peptides [15,16], angiotensin converting enzyme inhibiting peptides [14,17–19], renin inhibiting peptides [20] and antimicrobial peptides [19]. Moreover, PLS modeling has been applied to the study of functional properties of food proteins and polypeptides; these are studies where the functional properties under investigation are impacted mostly by proportion of relevant amino acids rather than sequence [21]. Despite the wide application in food science, there is dearth of information in the literature on the use of chemometrics approaches, such as PLS method, in studying bioactivity of food protein hydrolysates that contain mixtures of peptides. Previous chemometric work on food-related bioactive compounds involved PLS analysis to elucidate the structural requirements for potency of synthesized antioxidant polyphenols in different chemical, cellular and enzymatic oxidative systems [22–24]; these methods often resulted in the discovery of compounds with more potent end-point antioxidative activities.

**Figure 1.**
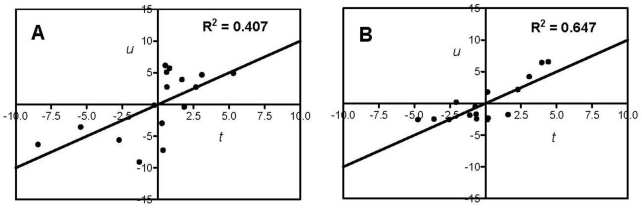

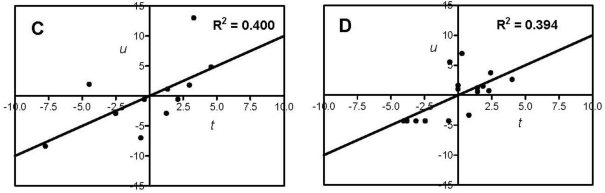
The *t/u* score plots of the partial least squares (PLS) models showing relationships between the antioxidant activity and amino acid descriptors (AA + gAA + Σ*z_i_*) of the food protein hydrolysates and peptide fractions; (**A**) DPPH radical scavenging; (**B**) ferric reducing antioxidant power (FRAP); (**C**) H_2_O_2_-scavenging, and (**D**) superoxide radical-scavenging.

PLS modeling of amino acid parameters (*X*) and four different antioxidant activities (*Y*) of the food protein hydrolysates and fractions resulted in 12 models as shown in Table 1. The ferric reducing antioxidant property (FRAP) model (AA only) gave the best fit and predictive power whereas models for H_2_O_2_-scavenging displayed the lowest predictive powers (Figure 1 and Table 1). The *R*^2^ values (Table 1) indicated that the PLS models explained 30% to 73% of the sum of squares in *Y*-variance for all the oxidative systems with up to 66% predictive ability (derived from *Q*^2^_cv_). The 12 models were theoretically validated initially by cross-validation during modeling and their predictive power also validated by permutation, where the bioactivity data were each randomly permuted a number of times but with unaltered *X*-variable followed by modeling of each permutation [13]. As shown in Table 1, repeated (20) rounds of permutation yielded cumulative *R^2^* (*R*^2^_cum_) intercept values of 0.006 to 0.314 and *Q*^2^_cv_ intercept values of −0.223 to −0.008 for the 12 models, which are within suggested valid limits of *R*^2^_cum_ intercept <0.4 and *Q*^2^_cv_ intercept <0.05 for valid PLS models [25]. The *t/u* PLS score plots show relationships between *X* and *Y* variables in the AA + gAA + Σ*z_i_* (antioxidant activity + amino acid group + sum of *z* values) models for the four oxidative systems (Figure 1A–D). With the exception of the weak fit for H_2_O_2_, the data showed good fit and must have contributed positively to obtaining valid models that we used to determine relationships between amino acids or groups of amino acids and contributions to antioxidant potential of the food protein hydrolysates.

**Figure 2.**
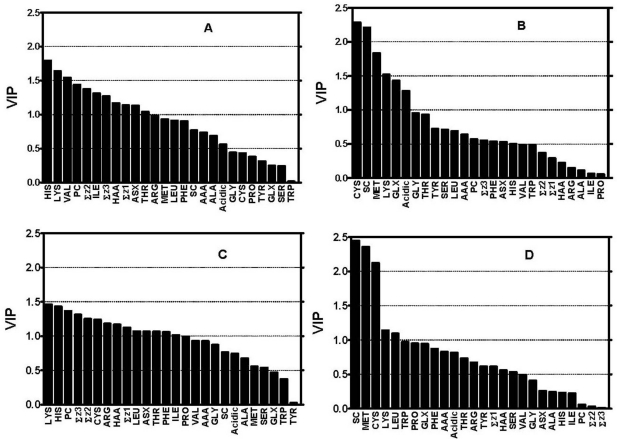
Variable Importance for the Projection (VIP) of the *3-z* scale models: (**A**) DPPH radical scavenging; (**B**) ferric reducing antioxidant power (FRAP); (**C**) H_2_O_2_-scavenging, and (**D**) superoxide radical-scavenging.

**Figure 3.**
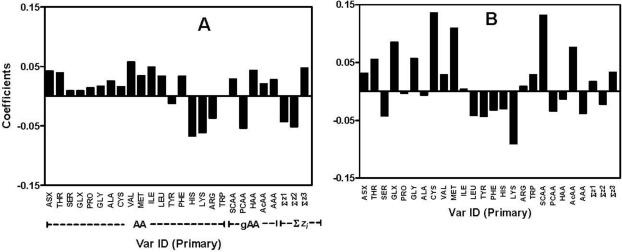

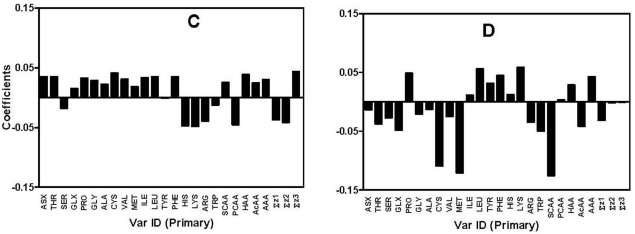
Coefficient plots of scaled and centered data of the partial least square regression models: (**A**) DPPH radical scavenging; (**B**) ferric reducing antioxidant power (FRAP); (**C**) H_2_O_2_-scavenging; and (**D**) superoxide radical-scavenging. The importance of a given *X*-variable is proportional to its distance (coefficient value) from the origin (zero). Above zero values indicate positive contributions while values less than zero indicate negative contributions.

The importance or relative contribution of the amino acid descriptor variables (*X*) in PLS modeling was obtained from the VIP plots (Figure 2A–D). The *X* variables with VIP > 1.0 are regarded as important with above average contribution while those with VIP < 0.5 are unimportant; variables with 1.0 > VIP > 0.5 could be important or not depending on the size of the dataset [26]. For example, the sulphur-containing (SC) amino acids as a group as well the individual amino acids that include *Met*, *Cys*, *Leu*, and *Lys* are strong contributors (VIP values > 1.0) to the superoxide model (Figure 2D). In contrast, *Gly*, *Ala*, *His*, *Asx*, and *Ile* are poor contributors to the superoxide model due to VIP values of <0.5. For our present study, *X*-variables were regarded as important strong contributors only when their VIP > 1.0 and weak contributors with VIP of 0.5–1.0. The relative contribution of *X*-variables in the PLS models depends on the value of the coefficients relative to the origin in the loading space [13]; in other words, the higher the coefficients in both directions, the more the contribution of the *X*-variable in explaining or predicting *Y* [27]. The coefficients (Figure 3A–D) and VIP plots (Figure 2A–D) indicated that the specific contribution of each or groups of amino acid and physicochemical properties (*3-z* scales) depends on the oxidative assay system. As shown in Figure 3A and Table 2, a high percentage composition of *Thr*, *Asx*, hydrophobic amino acids, as well as ↑Σ*z_3_* (high electronic properties), ↓Σ*z_1_* (low hydrophilicity or high hydrophobicity) and ↓Σ*z_2_* (low bulk/molecular size) of the samples contributed positively to 2,2-diphenyl-1-picrylhydrazyl (DPPH)-scavenging. Interestingly, PCAA including *His* (highest VIP value) contributed negatively to the scavenging of DPPH. A similar pattern was also observed for H_2_O_2_-scavenging in addition to the strong positive contributions of *Cys*, *Phe*, *Leu* and *Pro* in this oxidative system (Figure 3C). Moreover, the SCAA (*Cys + Met*) were observed to be the strongest contributing amino acids for ferric reducing antioxidant power of the peptide mixtures whereas high amounts of *Lys* strongly reduced this activity (Figure 3B). In contrast, *Lys* and *Leu* composition supported superoxide radical scavenging by the samples while SCAA *Met* and *Cys* (highest VIP values) strongly reduced this activity. Interestingly, low *z_1_* (high hydrophobicity) character seem to have strong positive contributions to scavenging of the free radicals like DPPH and superoxide anion radical (O_2_^−^) as well as H_2_O_2_ (a reactive oxygen species) but not FRAP. The results agree with previous reports that indicate hydrophobic amino acids are able to interact better (when compared to hydrophilic amino acids) with the lipophilic environments that contain these free radicals. Table 2 also shows other amino acid compositions and properties that weakly contributed positively or negatively to the antioxidant activities of the peptide samples in the four oxidative systems.

Due to heterogeneity of reactive species implicated in human disease conditions, the structure and composition of amino acid residues required for potent antioxidant activity by food protein hydrolysates and peptides could depend on the type of reactive species and reaction conditions, e.g., pH and solvent. Consequently, high levels of particular amino acid residues can potentially increase or decrease the antioxidant activity of food protein hydrolysates depending on the reaction environment. Amino acid residues are major physiological targets of oxidants leading to the formation of stable and unstable oxidation products [28]; thus, the rate of transfer of the radicals to amino acids and stability of the reaction products can substantially influence potency of amino acids as antioxidants. Plausible mechanisms of radical scavenging or quenching activity of amino acid residues of proteins and peptides include proton or hydrogen atom donation by amino acids such as *Tyr*, *Trp* and *Cys* using their phenolic, indolic and sulfhydryl hydrogen, respectively [1,7,10]. The electron-dense side chain groups of *His*, *Trp* and *Met* also contribute to antioxidant properties of proteins and peptides [28]. In our study, the food protein hydrolysates in the dataset were composed of varying amounts of amino acid residues, which positively or negatively impacted their antioxidant activities based on the PLS models.

The positive contributions of SCAA (*Cys* and *Met*) to the antioxidant activity of the samples (Table 2) can be attributed to their S-groups, which are prone to oxidation by the reactive species leading to the formation of stable oxidation products, cystine and methione sulfoxide, respectively [1,7,28]. The sulfhydryl group of *Cys* also acts as a strong reducing agent, hence its strong positive contribution in reducing ferric ion. Aspartic acid (*Asx*) and glutamic acid (*Glx*) can also donate their acidic hydrogen atoms near neutral pH, and this may have resulted to their strong contributions to the antioxidant activities of the samples. Similarly, *Phe* played a positive role in H_2_O_2_-scavenging and this can be attributed to susceptibility of its aromatic ring to oxidation. It is not clear how the HAA residues and low Σ*z_1_* (low hydrophilicity or high hydrophobicity) of the samples directly contributed to antioxidant activity of food protein hydrolysates and fractions, although HAA may have interacted with DPPH via hydrophobic forces thereby increasing the proximity of the radical to the active functional groups. Moreover, amino acids with high Σ*z_3_* exhibited positive effects in the assays except superoxide radical-scavenging, and this can be attributed to their electron density. Amino acids with less bulky structures (low Σ*z_2_*) correlated positively with antioxidant activities of the samples (Table 2), suggesting that steric effects can influence DPPH-scavenging activity of peptides, considering the bulky structure of the synthetic radical. On the other hand, the negative effects of the PCAA could be due to the fact that they can accept hydrogen ion near physiological pH and exist in the protonated state. Surprisingly, *His* residue was observed to negatively contribute to antioxidant properties of the peptide mixtures, based on the PLS models (Table 2). *His* can act as both hydrogen acceptor and donor near physiological pH; thus, its particular role in the complex peptide mixture could not be elucidated using these models. Moreover, the current work did not take into consideration the amino acid sequence of peptides, which could be important for *His*-containing peptides. Previously, the presence of *His* in synthetic peptides was reported to promote antioxidative activity in a linoleic acid oxidation model but not DPPH and superoxide radical-scavenging [8].

## 3. Experimental Section

### 3.1. *X*-matrix

The *X*-matrix contained data for individual 18 amino acids AA (*Asx* for *Asn + Asp* and *Glx* for *Glu + Gln*) and groups of amino acids (gAA) such as sulphur-containing (SCAA-*Cys* and *Met*), positively charged (PCAA-*Arg*, *Lys* and *His*), acidic (AcAA-*Asx* and *Glx*), aromatic (AAA-*Phe, Tyr* and *Trp*) and hydrophobic amino acids (HAA-*Pro*, *Ala*, *Cys*, *Val*, *Met*, *Ile*, *Leu*, *Tyr*, *Phe* and *Trp*). The gAA were used because amino acids within each group have certain similarities (e.g., presence of sulphur or aromatic ring, *etc.*), which allow PLS modeling based on group characteristics. The results can be used to determine for example, whether aromatic rings contribute a greater to certain antioxidant property than acidic or sulphur groups. The *X*-matrix data for the amino acids of 16 samples were presented as percentage composition (Table 3) and the groups (Table 4) as sums of the respective data. The amino acid data were derived from previous studies that fractionated hempseed and pea protein hydrolysates based on molecular size [29], hydrophobic property [5] and net cationic property [30]. The amino acid *3-z* scale was also used as *X*-variable in the multivariate descriptor matrix. The *3-z* scales was previously derived by Hellberg *et al*. [31] by principal component analysis from 29 physicochemical variables of amino acids and were interpreted to be related to hydrophilicity (*z_1_*), steric properties or side chain bulk/molecular size (*z_2_*) and electronic properties (*z_3_*) (see supplementary material). Algebraic sums of each of the *3-z* scores (Σ*z_i_*) were calculated for each sample (see supplementary material) as previously reported [21] using Equation 1.

(1) $$\sum z_{i}=\sum_{X=1}^{18}z_{iX}\;c_{X}$$

*X* represents each of the 18 amino acids in the protein hydrolysates and fractions, and *c* represents their percentage composition; the *z* values for *Asx* and *Glx* were calculated as averages of the *z* values of their respective constituent amino acids.

### 3.2. *Y*-matrix

The *Y*-matrix contained the antioxidant data for the various observations, specifically the ability of the food protein hydrolysates to scavenge nitrogen-centered 2,2-diphenyl-1-picrylhydrazyl (DPPH) radical, superoxide anion radical (O_2_^−^) and H_2_O_2_, and their ferric reducing antioxidant power (FRAP). These antioxidant data were chosen because of the physiological relevance of the reactive species (O_2_^−^ and H_2_O_2_) and reducing power in chronic human disease conditions, apart from DPPH-scavenging, which has been widely used in the literature as primary evaluation of antioxidant (reducing) capacity of food protein hydrolysates. The bioactivity data were presented as percentage scavenging of the free radicals and H_2_O_2_, and as absorbance at 700 nm for ferric reducing antioxidant power (see supplementary material). In order to ensure consistency in data interpretation, the antioxidant and amino acid composition data used in this study were reported by the same research group.

### 3.3. PLS Modeling

Modeling of the antioxidant activities (*Y*) as a function of the amino acid descriptors (*X*) of the protein hydrolysates was computed by the PLS method using SIMCA-P version 11.0 (Umetrics AB, Umeå, Sweden); the PLS models were generated for the four oxidative assay systems using individual (AA, gAA and Σ*z_i_*) and a combination of all the descriptors. All variables were centered and scaled to unit variance to ensure equal contribution in the models. The PLS models were validated theoretically using a combination of cross-validation and permutation tests [13]. The multiple correlation coefficient (*R*^2^) and cross-validation correlation coefficient (*Q*^2^_cv_) were computed by SIMCA-P software and used to represent model fit and predictive ability, respectively. The relative contribution of the amino acid (*X*) descriptors to the antioxidant activities of the samples was computed by the software and presented as the Variable Importance for the Projection (VIP) and coefficient plots.

## 4. Conclusions

The present work has shown that chemometrics approach using PLS models successfully elucidated specific contributions (positive or negative) of individual and groups of amino acid residues to the antioxidative properties of food protein hydrolysates; however, the effects depend on the oxidative assay system. Based on the PLS models, it was observed that previously reported antioxidant amino acid residues (especially *His*) had negative influence on the antioxidant activities (DPPH, H_2_O_2_, superoxide, ferric reducing) studied in this work. Overall, low hydrophilic property (high hydrophobicity) was a strong positive contributor to scavenging of free radicals (but not ferric reducing ability) by food protein hydrolysates. In contrast to previous assumptions in the literature, aromatic amino acids did not show strong contributions to antioxidant systems studied in this work (except for H_2_O_2_). However, the data cannot be used to preclude strong contributions of aromatic amino acids to other antioxidant systems that were not included in our report. This is because antioxidant activities of food protein hydrolysates could also be influenced by the amino acid sequence and interactions between neighboring residues; these factors were not part of the PLS analysis in this work. Data from this work can serve as background for further chemometrics study on bioactive food protein hydrolysates using larger uniformly generated datasets and determining the effect of amino acid sequence. Results from this study could contribute to proper understanding of the structure-function relationships of antioxidant food protein hydrolysates and peptides. The results could also enhance development of enzymatic tools and processing conditions that will concentrate antioxidant amino acids into highly potent fractions. Future studies to apply the results of this study may include optimization of enzymatic hydrolysis and processing conditions to enrich the final peptide product with the desirable amino acid residues. The choice of amino acids or proportion of amino acids that need to be dominant in potent antioxidant food protein hydrolysates will depend on the target antioxidant system.

## Supplementary Information



## Figures and Tables

**Table 1.** Summary of the partial least square regression models using amino acid compositions (AA), groups of amino acids (gAA) and sums of *3-z* amino acid scales (Σ*z_i_*) of the food protein hydrolysates. The multiple correlation coefficient (*R*^2^) estimates the model fit whereas the cross-validated correlation coefficient (*Q*^2^_cv_) indicates the models’ predictive powers.

**Antioxidant Property**	***N****^a^*	***X****^b^*	***A****^c^*	***R*^2^***^d^*	***Q*^2^_cv_***^e^*	**Permutation Test***^f^*	
						**Int.*R*^2^_cum_**	**Int. *Q*^2^_cv_**
DPPH-scavenging	16	AA + gAA+ Σ*z_i_*	1	0.407	0.327	0.241	−0.096
		AA	1	0.448	0.358	0.288	−0.108
		gAA	1	0.405	0.231	0.149	−0.043
		Σ*z_i_*	1	0.304	0.288	0.006	−0.127
Ferric reducing	16	AA + gAA + Σ*z_i_*	1	0.647	0.604	0.166	−0.208
		AA	1	0.728	0.668	0.223	−0.219
		gAA	1	0.536	0.531	0.073	−0.169
H_2_O_2_-scavenging	11	AA + gAA+ Σ*z_i_*	1	0.400	0.137	0.261	−0.122
		AA	1	0.467	0.136	0.314	−0.109
		Σ*z_i_*	1	0.340	0.232	0.088	−0.008
O_2_^−^-scavenging	16	AA + gAA + Σ*z_i_*	1	0.394	0.179	0.212	−0.063
		gAA	2	0.602	0.142	0.166	−0.223

a N, number of observations used for PLS analysis;

b *X*, *X*-variables (descriptors) in the validated PLS models;

c A, number of significant components used in PLS modeling;

d *R*^2^, multiple correlation coefficients;

e *Q*^2^_cv_, cross-validation correlation coefficients;

f Permutation test, *R*^2^_cum_ and *Q*^2^_cv_ intercepts were calculated by SIMCA-P software during model validation.

**Table 2.** Summary of contribution of amino acid compositions and properties to antioxidant activities of food protein hydrolysates based on partial least square regression coefficients and VIP plots of the AA + gAA + Σ*z_i_* models; strong contributors with VIP > 1.0 are shown in bold; (weak contributors with 1.0 > VIP > 0.5 are shown in brackets).

**Oxidative System**	**Positive Contributors**	**Negative Contributors**
DPPH radical scavenging	***Asp + Asn (Asx)***, ***Thr***, ***Val***, ***Ile***, **HAA***^a^*, ↑Σ***z_3_****^b^*, ↓Σ***z_1_****^c^*, ↓Σ***z_2_****^d^* (*Ala*, *Met*, *Leu*, *Phe*, *SCAA^e^*, *AcAA^f^*, *AAA^g^*)	***His***, ***Lys***, ***Arg***, ***PCAA****^h^*
Ferric reducing	***Cys***, ***Met*, *Glu + Gln (Glx)****,****SCAA***, ***AcAA*** (*Asp + Asp*, *Thr*, *Gly*, ↑Σ*z_3_*)	***Lys*** (*Ser*, *Leu*, *Tyr*, *Phe*, *His*, *PCAA*, *AAA*)
H_2_O_2_-scavenging	***Cys***, ***Phe***, ***Leu***, ***Ile***, ***Pro***, ***Thr***, ***Asx***, ***HAA***, ↑Σ***z_3_***, ↓Σ***z_1_***, ↓Σ***z_2_*** (*Gly*, *Ala*, *Val*, *Met*, *SCAA, AcAA*, *AAA*)	***His***, ***Lys***, ***Arg***, ***PCAA*** (*Ser)*
Superoxide radical scavenging	***Lys***, ***Leu*** (*Pro*, *Phe*, *Tyr*, *HAA*, ↓Σ*z_1_*)	***SCAA***, ***Met***, ***Cys*** (*Trp*, *Glx*, *AcAA*, *Thr*, *Arg*, *Ser*)

a HAA, Hydrophobic amino acids (*Pro + Ala + Cys + Val + Met + Ile + Leu + Tyr + Phe + Trp*);

b Amino acids with ↑Σ*z_3_* (electronic property) include *Cys > Pro > Asx > His > Trp*;

c Amino acids with ↓Σ*z_1_* (highly hydrophobic) include *Phe > Trp > Ile > Leu > Val > Met > Tyr*;

d Amino acids with ↓Σ*z_2_* (lower side chain bulk/molecular size) include *Gly > Val > Thr > Ala > Ile > Ser > Leu > Cys*;

e SCAA, sulfur-containing amino acids (*Met + Cys*);

f AcAA, Acidic amino acids (*Glx + Asx*);

g AAA, Aromatic amino acids (*Phe + Tyr + Trp*);

h PCAA, positively charged amino acids (*Lys + Arg + His*).

**Table 3.** The % amino acid (AA) composition of the food protein hydrolysates used as variables in the *X*-matrix for partial least square regression analysis.

**Sample ID**	***X*-variables (AA)**																	
	**ASX***^a^*	**THR**	**SER**	**GLX***^b^*	**PRO**	**GLY**	**ALA**	**CYS**	**VAL**	**MET**	**ILE**	**LEU**	**TYR**	**PHE**	**HIS**	**LYS**	**ARG**	**TRP**
1	11.39	3.68	4.63	20.06	4.00	4.29	4.47	1.32	4.66	1.81	3.84	6.75	3.45	4.60	2.78	2.97	14.07	1.23
2	9.49	3.60	4.73	15.18	3.19	3.23	4.91	0.29	5.67	1.94	4.15	9.91	4.78	7.68	2.61	3.19	13.87	1.58
3	11.70	3.77	4.79	19.31	4.04	3.93	4.77	0.66	5.26	2.03	4.16	7.26	3.50	5.01	2.47	2.94	12.96	1.44
4	12.79	4.01	4.69	22.71	4.23	4.54	4.30	1.26	4.45	1.85	3.98	5.15	3.06	3.21	2.47	2.56	13.60	1.16
5	12.70	4.00	4.47	22.87	4.89	4.71	4.12	1.58	4.24	1.80	3.90	4.82	3.62	2.85	2.49	2.51	13.31	1.11
6	13.79	3.60	6.20	13.92	5.15	3.76	5.01	0.24	5.63	0.91	5.43	9.91	3.87	7.41	1.61	6.10	6.83	0.68
7	13.94	3.89	6.63	17.12	2.33	3.52	5.54	0.18	5.23	0.70	4.13	8.70	2.77	3.97	2.49	9.07	9.79	0.00
8	10.63	3.86	5.71	14.78	6.47	5.00	4.30	0.39	4.45	1.70	4.04	6.68	5.33	7.76	3.28	7.35	8.00	0.27
9	12.59	3.34	6.19	13.75	5.14	3.96	5.03	0.39	4.13	0.87	6.71	9.95	7.15	8.73	1.90	4.26	5.15	0.74
10	10.85	3.11	4.41	12.87	5.42	4.66	3.44	0.38	5.82	1.07	5.85	14.57	5.09	12.03	1.81	3.31	3.97	1.36
11	11.04	3.22	3.82	6.64	8.05	3.26	3.62	0.29	7.68	0.68	9.13	19.48	2.44	16.44	0.63	1.20	1.22	1.16
12	15.09	4.43	5.16	20.30	5.16	4.12	5.47	0.41	4.77	1.07	4.99	10.16	3.41	6.81	1.05	3.40	3.12	1.06
13	11.23	3.42	3.96	22.14	5.78	3.83	4.31	0.26	4.28	0.88	3.23	6.55	4.12	4.50	3.55	8.03	9.32	0.63
14	9.86	3.59	5.73	10.81	4.56	3.47	4.62	0.13	3.64	0.96	3.21	8.22	3.21	6.20	3.76	16.38	10.19	1.46
15	8.12	2.68	3.72	9.47	2.37	3.87	4.18	0.12	2.66	0.51	2.25	8.92	5.61	4.86	4.22	11.79	24.50	0.14
16	6.61	1.74	4.24	9.04	2.93	2.00	2.48	0.11	2.02	0.21	2.14	6.64	3.48	2.24	4.26	16.76	30.18	2.93

a GLX = glutamine + glutamate;

b ASX = asparagines + aspartate.

**Table 4.** Amino acid groups (gAA) present in food protein hydrolysates used as variables in the *X*-matrix for partial least square regression analysis.

**Sample ID**	***X*-Variables (gAA)**				
	**SCAA***^a^*	**PCAA***^b^*	**HAA***^c^*	**AcAA***^d^*	**AAA***^e^*
1	3.13	19.82	36.13	31.45	9.28
2	2.22	19.67	44.10	24.67	14.05
3	2.70	18.37	38.14	31.00	9.95
4	3.11	18.62	32.64	35.50	7.42
5	3.39	18.31	32.95	35.56	7.58
6	1.15	14.54	44.24	27.71	11.96
7	0.88	21.35	33.55	31.06	6.74
8	2.09	18.63	41.39	25.41	13.36
9	1.26	11.31	48.84	26.34	16.62
10	1.45	9.09	55.03	23.72	18.48
11	0.97	3.05	68.97	17.68	20.04
12	1.48	7.58	43.32	35.39	11.28
13	1.14	20.90	34.53	33.37	9.24
14	1.09	30.33	36.21	20.67	10.88
15	0.63	40.51	31.63	17.59	10.62
16	0.33	51.19	25.18	15.65	8.65

a SCAA, sulphur-containing amino acids (*Met + Cys*);

b PCAA, positively charged amino acids (*Lys + Arg + His*);

c HAA, Hydrophobic amino acids;

d AcAA, Acidic amino acids (*Glx + Asx*);

e AAA, Aromatic amino acids (*Phe + Tyr + Trp*).
