# Supplementary material for: Chemometric Analysis of the Amino Acid Requirements of Antioxidant Food Protein Hydrolysates
Source: Int J Mol Sci. 2011 May 13;12(5):3148–61. doi: 10.3390/ijms12053148 (PMC3116181; doi:10.3390/ijms12053148)
Supplement: Supplementary file 1 [file ijms-12-03148-s001.pdf]

Chemometric Analysis of the Amino Acid Requirements of Antioxidant Food Protein Hydrolysates and Fractions

Chibuike C. Udenigwe, Rotimi E. Aluko

The Department of Human Nutritional Sciences and the Richardson Centre for Functional Foods and Nutraceuticals, University of Manitoba, Winnipeg, MB R3T 2N2, Canada

Supplementary Information

Table S1. The *3-z* scale of the 18 amino acids used for this study; the *3-z* values for Asx and Glx were calculated as averages of the *z* values of their respective constituent amino acids.

Amino acid	z_1	z_2	z_3
Ala	0.07	-1.73	0.09
Val	-2.69	-2.53	-1.29
Leu	-4.19	-1.03	-0.98
Ile	-4.44	-1.68	-1.03
Pro	-1.22	0.88	2.23
Phe	-4.92	1.30	0.45
Trp	-4.75	3.65	0.85
Met	-2.49	-0.27	-0.41
Lys	2.84	1.41	-3.14
Arg	2.88	2.52	-3.44
His	2.41	1.74	1.11
Gly	2.23	-5.36	0.30
Ser	1.96	-1.63	0.57
Thr	0.92	-2.09	-1.40
Cys	0.71	-0.97	4.13
Tyr	-1.39	2.32	0.01
Asx	3.43	1.29	1.60
Glx	2.63	0.46	-0.61

Table S2. Algebraic sums of the *3-z* scores ($\sum z_i$)[a] of the amino acids present in the 16 samples.

Sample ID	**X-variables ($\sum z_i$)**		
	$\sum z_1$	$\sum z_2$	$\sum z_3$
1	70.22	17.52	-49.21
2	12.23	19.53	-58.74
3	55.55	14.55	-48.72
4	93.66	15.57	-45.07
5	95.77	16.48	-40.96
6	14.76	-2.07	-33.66
7	80.91	4.40	-59.62
8	36.73	8.50	-36.49
9	-11.91	0.44	-21.31
10	-66.01	-9.92	-20.57
11	-162.17	-30.48	-5.77
12	20.55	-12.35	-14.42
13	81.51	23.39	-48.82
14	61.54	30.98	-75.06
15	94.62	62.87	-116.02
16	129.19	102.79	-146.66

[a] $\sum z_i = \sum_{X=1}^{\text{[illegible]}} z_{iX} c_X$

Table S3. Average values of the antioxidant data for the 16 samples used in the *Y*-matrix for partial least square regression analysis

Sample ID	% Radical scavenging activity (RSA)			$\Delta A_{700\ nm}$
	**DPPH**[a]	**Superoxide**	**H_2O_2**	**FRAP**[b]
1	2.97	0.00	NR[c]	0.0987
2	24.24	0.00	NR	0.0635
3	23.52	0.00	NR	0.0692
4	22.53	0.00	NR	0.1312
5	18.81	0.00	NR	0.1331
6	20.69	4.51	61.58	0.0126
7	14.25	25.22	40.45	0.0020
8	9.72	28.12	51.09	0.0000
9	13.80	26.15	57.20	0.0100
10	18.77	27.52	63.28	0.0300
11	22.28	32.82	71.21	0.0400
12	21.87	46.58	92.48	0.0116
13	0.00	53.42	57.17	0.0035
14	5.52	23.20	51.06	0.0000
15	8.76	38.13	63.67	0.0012
16	4.40	23.92	36.85	0.0000

[a]DPPH, 2,2-diphenyl-1-picrylhydrazyl radical
[b]FRAP, ferric reducing antioxidant power
[c]NR, data not reported in the literature
